# Supplementary material for: Age-preserved semantic memory and the CRUNCH effect manifested as differential semantic control networks: An fMRI study
Source: PLoS One. 2021 Jun 15;16(6):e0249948. doi: 10.1371/journal.pone.0249948 (PMC8205163; doi:10.1371/journal.pone.0249948)
Supplement: S1 File — (DOC) [file pone.0249948.s001.doc]

April 3, 2021

**Object:** Data Availability Statement

Dear Editors of PLOS ONE,

The authors will comply with the Centre de Recherche Institut Universitaire de Gériatrie de Montréal (CRIUGM) Ethics Committee and the Centre intégré universitaire
de santé et de services sociaux du Centre-Sud-de-l'Île-de-Montréal requirements (CÉR-VN: Comité d’Éthique de la Recherche- Vieillissement et Neuroimagerie), the Canadian Institutes for Health Research requirements (https://cihr-irsc.gc.ca/e/29072.html), the minimal data policy of PLOS One to allow for replication of all study findings reported in the article, as well as related metadata and methods, including :

- The values behind the means, standard deviations and other measures reported;
- The values used to build graphs;
- The points extracted from images for analysis.

Wherever relevant data and information are not shared at the Registered Report Protocol submission, they will be shared by the time the Registered Report (2^nd^ stage) is submitted. We will share the preprocessed functional datasets in MNI space publicly in Open Science Framework (https://osf.io/) with a digital object identifier (DOI) to permanently identify the dataset (Nichols et al., 2016), and we will index it in the Canadian Open Neuroscience Platform (https://conp.ca/) to increase findability. All related data (stimuli, instructions, ethics’ approval) are in the osf.io platform (doi: 10.17605/OSF.IO/F2XW9). In addition, once they become available, we will upload our unthresholded statistical maps to neurovault (https://neurovault.org/), an online platform sharing activation data. Permanent links to the unthresholded statistical maps to be uploaded at Neurovault will be provided as part of the dataset deposited on the OSF, under the same DOI (DOI: 10.17605/[OSF.IO/F2XW9](http://OSF.IO/F2XW9)). Though the authors intend to make their raw data publicly available, ethical regulations at our institute do not allow for sharing of raw data at the moment, due to privacy risks for the human subjects and risk of re-identification (data contain potentially identifying information). These will remain stored in a private server, accessible on demand and following ethics committee approval. Data access requests can be made at:

**Unité de neuroimagerie fonctionnelle (UNF)**

https://unf-montreal.ca/contact-us/
Centre de recherche de l’institut universitaire de gériatrie de Montréal
4565 Queen-Mary road
Montreal, QC
H3W 1W5

**Administrative Contact**Émilie Dessureault
514-340-3540 ext. 3633

[emilie.dessureault@criugm.qc.ca](mailto:emilie.dessureault@criugm.qc.ca)

and

**Ethics Committee of CRIUGM (Comité d’Éthique de la Recherche du CRIUGM)**

<http://www.criugm.qc.ca/en/the-research/ethics.html> and

<https://ciusss-centresudmtl.gouv.qc.ca/mission-universitaire/ethique-de-la-recherche>

66 Sainte-Catherine est
Montréal (Québec) H2X 1K6
Courriel : [karima.bekhiti.ccsmtl@ssss.gouv.qc.ca](https://ciusss-centresudmtl.gouv.qc.ca/mission-universitaire/karima.bekhiti.ccsmtl@ssss.gouv.qc.ca)
Téléphone : 514 527-9565, poste 3223

With kind regards,


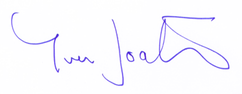


**Yves Joanette, Ph.D., FCAHS**

Professor, Faculty of Medicine, University of Montreal

Lab Director, CRIUGM

Deputy Vice-Principal Research, University of Montréal

Member & Former Chair, World Dementia Council
